# Patient-centred orientation of students from different healthcare disciplines, their understanding of the concept and factors influencing their development as patient-centred professionals: a mixed methods study

**DOI:** 10.1186/s12909-019-1787-4

**Published:** 2019-09-11

**Authors:** Sheeba Rosewilliam, Vivek Indramohan, Richard Breakwell, Bernard Xian Wei Liew, John Skelton

**Affiliations:** 1 School of Sports, Exercise and Rehabilitation Sciences, University of Birmingham, Edgbaston B15 2TT, Birmingham, UK; 2 Department of Life Sciences, School of Health Sciences, Faculty of Health, Education and Life Sciences, Birmingham City University, Birmingham, UK; 3 School of Nursing, University of Birmingham, Birmingham, UK; 4 School of Sport, Rehabilitation and Exercise Sciences, University of Essex, Colchester, Essex UK; 5 Institute of Clinical Sciences, University of Birmingham, Birmingham, UK

**Keywords:** Patient-centred care, Clinical placement, Health professional education, Mixed methods

## Abstract

**Background:**

A patient-centred approach to care is increasingly the mandate for healthcare delivery. There is a need to explore how health professional students develop patient-centred attributes. This study aims to understand the extent of patient-centred orientations of health professional students, their perceptions and factors influencing their adoption of the approach.

**Methods:**

The study used a cross-sectional, parallel mixed methods design combining a survey using the Patient-Practitioner Orientation Scale (PPOS) followed by focus groups with medical, nursing, physiotherapy and speech and language therapy students. Data included students’ age, gender, programme, and placements experienced. Pearson’s chi squared and the non-parametric equivalent Kruskal-Wallis H test were done to test for differences in demographics for appropriate variables. One-way ANOVA or Welch test was done to explore differences in PPOS scores. Regression analysis was done to test the influence of the demographic variables on PPOS scores. Data from focus groups were coded, categorised and organised under themes appropriate to the research aims.

**Results:**

Of the 211 complete responses, significant differences were observed between medical and physiotherapy students in total PPOS scores, (MD -8.11 [95% CI -12.02 - 4.20] *p* = 0.000), Caring component (MD -4.44 [95% CI - 6.69, − 2.19] p = 0.000) and Sharing component (MD -3.67 [95% CI -6.12 -1.22] *p* = 0.001). The programme in which students were enrolled i.e. Medicine and SALT were the only indicators of higher PPOS total scores (F = 4.6 Df 10,69; *p* = 7.396e-06) and caring scores (F = 2.164 Df 10, 69 *p* = 0.022). Focus groups revealed that students perceived patient-centredness as holistic yet individualised care through establishing a partnership with patient. They identified that their student status, placement pressures, placement characteristics especially mentoring influenced their development of patient-centred attributes.

**Conclusion:**

This study highlights the fact that the pressures of training in the National Health Service affects the development of students’ patient-centred orientation. There is a need for further work to explore aspects related to mentor training, for the development of patient-centred attributes, in a curricular framework structured on students’ needs from this study.

**Electronic supplementary material:**

The online version of this article (10.1186/s12909-019-1787-4) contains supplementary material, which is available to authorized users.

## Introduction

A target-oriented culture has resulted in poor quality of care in many National Health Service (NHS) trusts - “a culture focused on doing the system’s business - not that of the patients” [1] pg 4, [2, 3]. A key issue is the limited adoption of a patient-centred approach to care delivery [4–6].

Patient-centredness is a multi-faceted concept, including, it has been suggested, communication, collaboration, respect, therapeutic relationship between parties, consideration of care for all aspects of health (including psychological and social needs) [7]. The case has been widely made for the benefits of patient-centred care in terms of patients being satisfied with the clinician [8], having better emotional health [9], better recovery from discomfort, fewer concerns and fewer referrals [10, 11]. However, the culture and attitude of healthcare professionals for adopting patient-centred care within many hospitals seems to be defensive and resistant to change [12]. This points to a need to develop a future workforce who are patient-centred to improve quality of care in our health services.

Professional behaviours are cultivated and developed primarily when clinicians are students. A wide range of curricular initiatives have been claimed as promoting patient-centredness, including inter-professional programs [13–15], ‘Physicianships’ that integrate bio-psychosocial content with moral and behavioural attributes [16], service learning opportunities [17], extended community based learning [18] and the use of virtual patients [19]. Moreover, it has been identified that certain factors such as being female, having a religious background, the content of the curriculum and having community placements positively influenced students’ patient-centred orientation amongst medical and nursing students [20–24]. However, it has also been found that patient-centred attitudes eroded in nursing and medical students in the later years of their training as they became more clinician-centred [24–26]. It is possible that aspects of the hidden curriculum [27] may serve to undermine or confirm the intentions of the curriculum designer.

Health professional students based in different educational philosophies, work closely in multidisciplinary teams and potentially learn from each other. However, it is not clear whether their enrollment on different programmes, clinical exposure, or age influences students’ patient-centred orientations. Moreover, interpretations of the patient centredness concept are likely to vary with context [28] and it can be difficult to decide just how to enact it in particular situations [21]. In the current educational climate where different health professional students work and learn together on clinical placements, it is important to gain an understanding of their shared perceptions about patient-centred approach and factors that influence their delivery of patient-centred care (PCC). Knowledge of the healthcare professional students’ shared understanding about PCC is foundational to build curricular frameworks and clinical training strategies that students would engage in, for their development as patient-centred clinicians. This study intended to investigate the perceptions of students from medicine, nursing, physiotherapy and speech and language therapy (SALT) as they are key professions within a multidisciplinary team.

### Aims and objectives

The aim of this study is to explore the patient-centred orientation of students from different health disciplines and whether the key variables of age, gender, programme of study or placement exposure predicted their patient-centred orientation scores. A secondary aim was to explore students’ shared perspectives regarding their understanding of a patient-centred approach and factors that influenced their development as patient-centred professionals during their training period.

## Methods

The study adopted a cross-sectional, parallel mixed methods design involving both quantitative and qualitative approaches [29] . The study design involved surveys using questionnaires and focus groups [30] based on content analysis [31]. Mixed methods were used to gain holistic perspectives within this area and to complement the data collected from each of the methods. The population of interest were students in various health professional courses such as medicine, SALT, physiotherapy, occupational therapy and nursing to represent the wide membership of a multi-disciplinary healthcare team. Three universities across West Midlands (United Kingdom) that delivered health professional courses were approached for their participation in this study. The sampling strategy was purposive, and criterion based. Inclusion criteria were that the students are registered on any of the above listed professional courses, of both gender and willingness to participate. They should have done a minimum of two placements so that experiences from these placements can be explored. To standardise across programmes, third year students from all the above courses were invited to participate in the first part of the study. Third year was the final year of study for physiotherapy, nursing and SALT students. The final (fifth) year medical students were approached, but due to proximity of examinations a few students only volunteered to participate in the qualitative arm of the study.

The students were approached at the end of a whole group lecture by a tutor from a different programme to avoid coercion and participation bias. Students were given information sheets detailing the purpose and methodology of the study. They were then asked to complete the paper-based questionnaire and a consent form for completing the questionnaire and participation in the second part of study which used focus groups (FG). To increase response rate for the survey, an online version of the same questionnaire was emailed to the students but failed to get extra responses. Those who gave consent were invited for the FG which were conducted later in a room within the relevant University. For the focus groups, sample sizes were representative of each programme. Ethical approval was granted by the Science Technology Engineering and Mathematics ethics committee at the researchers’ University (ERN_17–0413).

The questionnaire collected information regarding their demographics such as their programme, year of education, the number of placements and details of placements. Their patient-centred attitudes were measured using the Patient Practitioner Orientation Scale (PPOS) a valid and reliable outcome measure that has been previously used with students from different healthcare disciplines such as medicine, physiotherapy, nursing and SALT [20, 24, 32, 33]. The PPOS has 18 items with a six-point Likert (strongly agree-strongly disagree) to score nine-items each for Sharing (belief that patients desire information) and Caring. The total scores for all items (range18–108), scores for caring (range 9–54) and sharing (range 9–54) were calculated and mean scores of items for caring, sharing and total were calculated (range1–6) [8]. The placement details entered by students were categorised into five categories Paediatric, Mental Health, Physical rehabilitation, Acute and Specialisms and Community and Hospices based on the setting and patient population seen. The specific placements within these categories are given in Additional file 1.

Focus groups were conducted using a question guide (Additional file 2) for self-selected groups of students from within these participating cohorts, to gain an understanding of their knowledge about this concept, its application and the facilitators and challenges from their learning experiences. Focus groups were used as they were time-efficient to gather perceptions and learning experiences, yet, collected information that was moderated within groups and hence was collective knowledge. The focus group guide (Additional file 2) was piloted in two groups of physiotherapy students from a previous cohort. The questions in the guide explored understanding and importance of the concept, challenges and facilitators to being patient-centred, and teaching and training about being patient-centred within university and on placements. Each focus group was conducted by tutors from a programme different to that the students were enrolled in. A scribe also a tutor from a different programme was present at each FG meeting. All researchers involved in conducting FG and analysing data had a master’s qualification, had previous experience in conducting qualitative research involving FG and were educators interested in improving patient-centred curriculum.

### Analysis

The quantitative data from the questionnaire and the sample characteristics such as age, gender, programme and placements experienced were summarised using descriptive measures using SPSS software (Statistical Package for the Social Sciences, version 21(C) Copyright IBM® Corp). The demographic variables were not normally distributed. Appropriate inferential tests were carried out on the data to answer the research question [34]. Tests for differences between programmes were carried out using Pearson’s chi squared or the non-parametric equivalent Kruskal-Wallis H test. Post hoc tests for direction of differences were explored using Dunn’s test with Bonferroni correction for age and number of placements. Following descriptive analysis of the PPOS scores for caring, sharing and totals, one-way ANOVA or Welch tests were used to explore differences in these scores between these groups depending on their distribution and homogeneity. Post hoc tests to determine direction of differences included Tukey’s test or Games Howell’s test (when Levene’s test for homogeneity showed *p* < 0.05.). Regression analysis was carried out to see if any of the demographic variables influenced the patient-centred orientation scores for caring, sharing or both.

Focus groups were recorded using digital recorders. The FG discussions were closed with a summary to the students and when they had no additional views to express. No repeat discussions were arranged, and data was not sent back to students for verification. The tapes were transcribed using external resources. The verbatim transcriptions were analysed using coding for content analysis [31]. Each FG transcript was independently coded and analysed by two researchers. Thus at least two members of the team coded each of the transcripts. They met to discuss the common categories and the overarching themes derived from each FG. These meetings served to clarify themes and enrich interpretations that were derived by the primary analyst. The first author was present for all of the meetings to seek clarification and summarise discussion between analysts. Once all the FG data were analysed the key themes across all FGs were synthesised by the first author. The final year medical students invited to participate were busy with their exam preparation and did not participate in the survey but came forward for the focus groups. However, their focus group data is not reported here as we could not relate their views to their level of patient-centred orientation (which is a key aim for mixed methods studies). Moreover, we could not compare their patient-centred views with other groups without an understanding of their extent of patient-centred orientation.

Synthesis of the findings from the quantitative and qualitative components involved highlighting the statistically significant findings, common and salient features to compare information presented from each of the disciplines [35].

## Results

Across the three universities approached, one of the programmes refused participation. Within the remaining two universities, four programme leads agreed to participate. Of the 470 survey questionnaires given out, 215 questionnaires were completed and 211 had complete demographic data and were included in the analysis. The response rate was 40% (*n* = 28/70) for nurses, 29% (*n* = 86/300) for medical students, and 100% each for Physiotherapy (*n* = 47/47) and SALT (*n* = 50/50) students. The numbers and characteristics of participants such as age, gender and placement data from different programmes are shown in Table 1.

**Table 1:** Participant characteristics and differences between groups

Characteristic	1. BSc Physiotherapy *n* = 47	2. MBChB *n* = 86	3. BSc Nursing *n* = 28	4. SALT *n* = 50	Overall	Tests for differences
No. (%)	47 (22%)	86 (41%)	28 (13%)	50 (24%)	211 (100%)	
Gender						
M	11	15	2	0	28 (13%)	*p* = 0.002 ^#^
F	33	68	25	50	176 (83%)	
Age Mean (SD)	21.5 years (2.4)	21.4 years (1.4)	24.7 years (7.9)	24.9 years (6.8)	Range 20–52 years 22.7 years (4.9)	*P* = 0.001^∞^
Age-range < 24 years Mature students > 24 years	40	73	18	28	159 (80%)	*p* = 0.000 ^#^
	3	9	8	21	41 (20%)	
Number of Placements Mean (SD)	5.95 (0.2)	2.78 (1.2)	8.60 (1.0)	2.29 (0.46)	4.02 (SD 2.5)	*P* = 0.000^∞^
Range of placements						
1–3 placements		64	–	50	*n* = 114 (54%)	
4–6 placements	47	20	1	–	*n* = 68 (32%)	
7–10 placements	–	–	25	–	*n* = 25 (12%)	
Type of Placement						
Had Paediatric placement	31	0	22	41	94 (45%)	*p* = 0.000^#^
Had Mental Health placement	13	0	19	5	37 (18%)	*p* = 0.000^#^
Had Physical Rehabilitation placement	19	0	0	1	20 (10%)	*p* = 0.000^#^
Had Acute and Specialties placement	47	84	26	28	185 (88%)	*p* = 0.000^#^
Had Community and Hospices placement	30	28	19	16	93 (44%)	*p* = 0.000^#^
PPOS Caring total mean (SD)	32.11 (5.2)	36.55 (3.6)	32.89 (5.1)	35.84 (5.7)	34.91 (5.1)	Welch test Stat = 11.28 Df = 3,79.86 *p* = 0.000 *
Caring-average of 9 items	3.56	4.06	3.65	3.98	3.87	
Sharing Total mean (SD)	36.55 (5.66)	40.22 (4.4)	37.32 (6.4)	38.94 (5.4)	38.72 (5.4)	One-way Anova
Sharing - average of 9 items	4.06	4.46	4.14	4.32	4.30	F = 5.770 *p* = 0.001 **
Total Scores mean (SD)	68.66 (9.07)	76.77 (6.29)	70.21 (9.06)	74.78 (9.65)	73.62 (8.81)	Welch test Stat- 12.11 Df= 3, 79.99 *p* = 0.000 ***
Average of 18 items	3.81	4.26	3.90	4.15	4.09	

Tests for differences

^‡^ Post Hoc Dunn’s with Bonferroni correction for Age *p* = 0.005 between 1 and 4 and 2 and 4; For Number of placements p = 0.001 between 4 and 1,

4 and 3, 2 and 1, 2 and 3

*Games-Howell 1.00 and 2.00 MD = −4.44 SE = 0.86, P = 0.000, CI (− 6.69–2.19); ** Tukey’s HSD 1.00 and 2.00 MD = −3.67, SE = 0.95 *p* = 0.001, CI (−6.12

-1.22); ***Games-Howell 1.00 and 2.00 MD = −8.11, SE = 1.49, *p* = 0.000, CI (−12.02 -4.20)

All demographic variables were not normally distributed (Shapiro-Wilks *p* = 0.00) and were significantly different in the student groups from various programmes (Pearson Chi squared & non-parametric equivalence Kruskal-Wallis H test, *p* < 0.05). Tests for differences were significant for age between SALT and medical and SALT and physiotherapy students. There were significant differences in the number of placements between SALT and physiotherapists and nurses and between medical students and physiotherapists and nurses (Table 1).

**Fig. 1:**
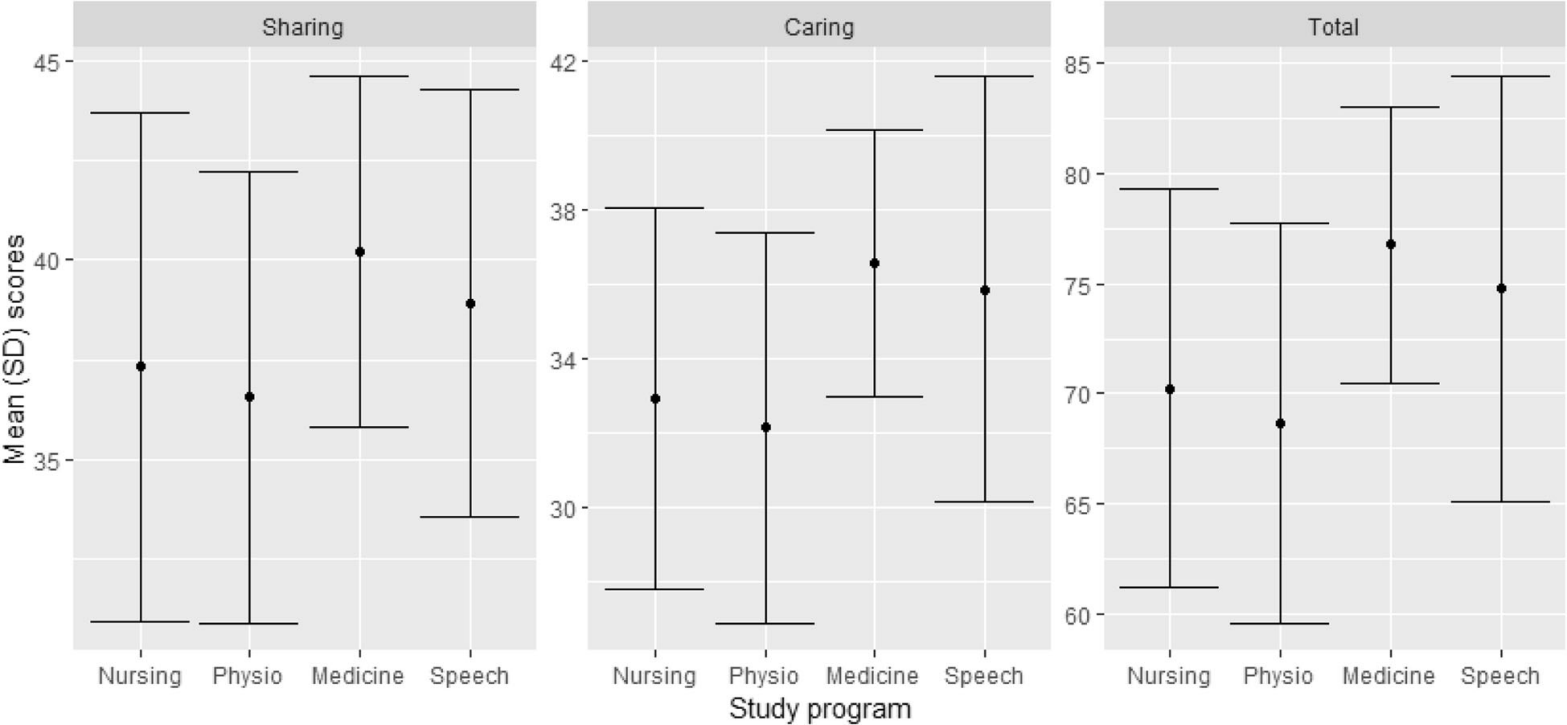
Distribution of Sharing, Caring and Total PPOS Scores

The overall total PPOS scores averaged at 73.62 (SD 8.81) with an average of 4.0 for the 18 items assessed. The differences between groups in the PPOS scores are shown in Table 1. The physiotherapists scored the lowest average of 68.66 (SD 9.07; item mean 3.81) and the medical students scored the highest with an average of 76.77 (SD 6.29; item mean 4.26) (Fig. 1). The medical students scored higher than physiotherapy students in total PPOS scores, with a mean difference of − 8.11 which was statistically significant (*p* = 0.000, 95% CI -12.02 - 4.20). For the Caring component the participants scored an average of 34.91 (SD 5.1, mean item score 3.87). The medical students scored higher than physiotherapy students in PPOS Caring component with a mean difference of − 4.44 which was significant (*p* = 0.000, 95% CI - 6.69 -2.19). Likewise, in the Sharing component, students had an average score of 38.72 (SD 5.4) and a mean item score of 4.30. The medical students scored higher than physiotherapy students in this Sharing component with a mean difference of − 3.67 which was significant (*p* = 0.001, 95% CI -6.12 - 1.22).

The multiple regression analysis (Additional file 3) included 200 participants’ data with complete data sets and showed that the programmes medicine and SALT were the only indicators of higher PPOS total scores (F = 4.6 Df 10,69; *p* < 0.001) and caring scores (F = 2.164, Df 10, 69 *p* = 0.022).

### Qualitative findings

**Fig. 2:**
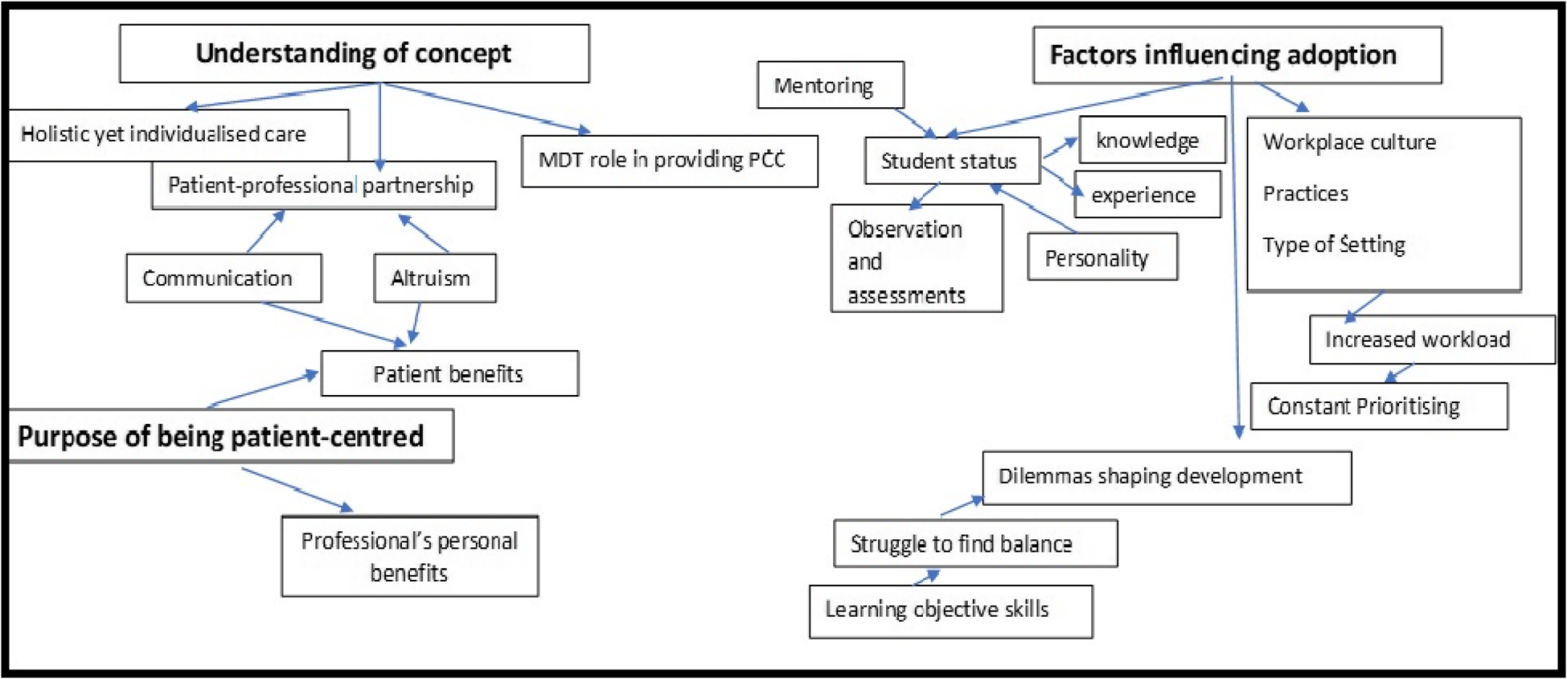
Qualitative findings presented as Themes and Categories

Twenty-one students from the questionnaire phase participated in four focus groups. Their characteristics are outlined in Table 2. The sample size and durations of the groups were: medicine (*n* = 3) for 44 mins, physiotherapy (*n* = 7) for 76 mins, SALT (n = 7) for 27 mins, nursing (*n* = 4) for 93 mins. The qualitative themes drawn from the content analysis describes the students’ understanding of patient-centred care approach and the factors influencing the development of patient-centred skills across the disciplines. The key themes derived from the data are represented in Fig. 2, following which they are explained with illustrative quotes.

1. Understanding of concept:

**Table 2:** Characteristics of students who participated in the focus groups

No	Programme	Year of Study	Gender	Number of Placements	Age
1.	SALT *	3	F	2	23
2.	SALT	3	F	2	35
3.	SALT	3	F	2	42
4.	SALT	3	F	2	22
5.	SALT	3	F	2	–
6.	SALT	3	F	2	28
7.	SALT	3	F	2	29
8.	Physiotherapy	3	M	6	20
9.	Physiotherapy	3	F	6	20
10.	Physiotherapy	3	F	6	20
11.	Physiotherapy	3	F	6	21
12.	Physiotherapy	3	F	6	21
13.	Physiotherapy	3	M	6	21
14.	Physiotherapy	3	F	6	25
15.	MBChB **	3	F	2	21
16.	MBChB	3	F	2	21
17.	MBChB	3	F	2	20
18.	Nursing	3	F	9	28
19.	Nursing	3	F	9	22
20.	Nursing	3	F	8	21
21.	Nursing	3	F	9	21

*SALT- Speech and Language Therapy; **MBChB - Bachelor of Medicine and Bachelor of Surgery

Students expressed a broad understanding of the approach which included aspects related to holistic yet individualised care, patient-professional partnership and engaging multi-disciplinary teams in providing PCC.

- Holistic care was perceived as the use of a tailored approach to meet individualistic needs. Understanding of personal situation, beliefs, values, emotions and needs were suggested as a precursor to building personalised care. Patient-centred values such as being empathetic and considerate were suggested as morals.

*“taking their values into consideration and their beliefs. Like taking the whole picture and the story of the patient into consideration … I think patient-centred care is individualised … I think like the main principles of patient-centred care, and empathy and taking in people's considerations, it's more - before uni [university] though that's just like pure morals”* Physiotherapy FG

b) Students perceived that to be patient-centred, professionals should communicate effectively, give information and involve them in shared decision making, thereby building a partnership with patients. A key process for enabling the above was mentioned as the goal-setting process to set and address goals that linked with patient outcomes.

*“what they personally want with their goal is important rather than what we may want personally as their goal … Yea so they are involved in the decision-making process and like the choices to be an equal partner … and we have to facilitate this by providing them with accessible information on their condition”* SALT FG

Communication was perceived to involve listening to patients about their clinical needs to decide on treatment. All student groups mentioned open communication that is honest and transparent and to the extent of sharing information about availability of resources or the lack of it. Medical students mentioned listening as a skill to develop good rapport.

*“I feel as though patient-centred care is listening to the patient, taking their values into consideration and communicating to them as equally as you'd communicate to the MDT. It's really important you've got to be open and transparent to try and gain that trust with the patient.”* Physiotherapy FG

*“So if you don't rush off when they start talking about their grandchildren … , then if you stay and listen to that then, in the future, you'll have a much better rapport with them.”* Medical FG.

c) The MDT approach was mentioned by all the groups to play a key role in delivering PCC through discussing patient information within team and the role of patient advocate. Nurses stressed family involvement and suggested that nurses were the link for patient, family and MDT to deliver PCC.

*“The families help you to identify their needs a lot of the time because the families will say.*..*it makes a difference to have the family … We get that contact with them (patient), that the other professionals don't but, … having more exposure to working with them gives more of a chance to … So we can act as their advocate, as the patients' advocates. I think all healthcare professionals are but … , we're just there all the time”* Nursing FG

2. Purposes for being patient-centred:

Students stated there were benefits both to patients and themselves. They believed that PCC gives them the opportunity to deliver what patients want, gain their trust and build rapport with patients. Though all four groups suggested, it improves compliance with treatment bringing about better outcomes for the patient, there were differences in their reasons for being patient-centred as described below. The Physiotherapy group were specifically focused on goal-setting and empowerment to ensure patients take control of managing their condition as the aim of physiotherapy is to improve patients’ independence.

*“I think like a patient/physio relationship makes them have more trust in you … makes you seem a bit more trustworthy as in like you've got their best interests at heart. They're more likely to comply with what you've asked them to do. The small short-term goals, they're patient-centred care in that they give them a sense of achievement and a sense of direction. They're feeling like they are actually in control of their health and they are achieving things.”* Physiotherapy FG

Nursing students reported that patient-centred care saved time in some instances, gained them appreciation from patient and family, and gave them a sense of satisfaction and happiness from their jobs. Medical students described it as the fun part of being a clinician, whereas Physiotherapists mentioned patient consent, a part of PCC, as a legal requirement and patient-centred attributes were employable skills. Students described wanting to help patients, build relationships and rapport, do kind acts and decent gestures to make patients feel that they are cared for. Students suggested patients would be better satisfied, grateful, less distressed and happy with care.

*“I think as a nurse as well knowing that and families especially makes you feel like you're doing a good job as well. Everybody's happy in an ideal world, but yes, I think it definitely makes you feel more competent as well, like, yes, okay. I mean you have your good days and bad days but just little comments, for me anyway like one big comment I'm just like, 'Oh yes', said I was a good nurse … Your patient's happy, you're happy.” Nursing FG*

*“Nowadays, when so many things depend on like imaging, or different diagnostic tests and stuff, it's so important to keep the only fun part of it, where you actually get to interact with a patient nicely. I think that's like a real doctory part of the job”.* Medical FG

*“I think, for me, like I came into the profession because I wanted to help people and that's what I wanted to do … that tied back, like the relief on her face because she was hot and clammy and she just really appreciated it, which was nice … just to know to call the patient what they'd like to be called the patient is immediately much more relaxed and responsive”* Nursing FG

3. Factors influencing development of PCC attributes

Students reported that their limited knowledge, roles and dilemmas they faced on their placements shaped their development as patient-centred professionals. They also attributed their patient-centred practice behaviours and learning to intrinsic factors such as their perceived status and certain external influences. Factors influencing their learning experiences are discussed below.

- Students reported limited knowledge due to limited prior practical experience influenced their PCC. Their major focus had been on developing knowledge of physical aspects or bio-medical knowledge till they came on to placements. They felt that teaching and practice were not linked in some areas such as when assessments focussed on skills and targets.

“*It sometimes can be difficult in practice when we're very much taught to be physical health nurses and I think sometimes the other things can be a little bit of an add-on”* Nursing FG

*“ 'Oh, I haven't done three bloods yet', they (students) would go specifically to a ward and ask the nurses and doctors if they had any patients for bloods that needed taking, whereas - I don't know, because that's not really patient-focused, but just trying to get something out of them, like you want their blood rather than to actually give them care.”* Medical FG

b) Students claimed that personal anxieties about their status as a student inhibited expression of patient-centred behaviours such as acting on patient’s concerns. When talking to patients, they were concerned whether patients would reciprocate or even co-operate with a student. They reported being afraid to approach patients or senior clinicians.

*“I mean, when I got to hospital, I was just scared to talk to doctors, basically. I thought everyone was so busy I didn't want to interrupt them to ask questions or anything like that, because they had lots going on, they didn't have time to talk to me. Then also approaching patients, I was like, well, I don't see why they'd want to talk to us because we can't really help them, you know”* Medical FG

Contrarily, some students felt that they had more time to spend with patients due to lesser responsibilities, said that patients find them more approachable than busy clinicians. They tend to spend more time doing elaborate assessments as students and work hard to earn patients’ trust.

*“So you do just pop your head into the bay, say, 'Hello,' to people. Then they do feel you've got that time to chat to them I think, which maybe once you're qualified you don't always have that spare time … going back to the trust thing, to talk to them a lot more to gain their trust. Like you'd go into more detail, like you get more of a social history, and I think because you're getting that social history out of them and you're investing time in them.”* Physiotherapy FG

c) Students claimed that external factors such as being observed, assessed or dominated by clinicians who served as their educators inhibited their natural exhibition of patient-centred behaviours. Due to the power differences, even in instances where there were negative role models, students did not want to upset the mentor and had to agree to the clinician’s approach to care. Lack of positive role models was suggested in all health care professions, but, specifically the medical students highlighted that senior clinicians were very bio-medical and so students tended to look to junior clinicians as role models to be more patient-oriented. Hence behaviour of current NHS clinicians was a key factor affecting students’ learning of patient-centred attributes.

*“I'd say a lot of the time, like as a student, it's if you're being assessed. If you're being watched by someone or you're very conscious that they're like expecting you to maybe do certain things to meet the assessment criteria,”* Physiotherapy FG.

*“I think that's the main problem with, you're not quite happy with how things are in practice and your mentor is marking you, there's no escaping that, really,*” Nursing FG

*“I mean, you see good examples and bad examples, but from what I've seen, like the more senior consultants tend to be the ones who are most in a rush, or don't stop to let people ask questions and things, and I think that's probably part of their job in terms of they've got the most pressure on them and things … ”* Medical FG

The other common factors that were mentioned by the students were the type of setting and work culture where they were placed and status of patients such as complexity of the patient’s condition and their personalities.

*“I think perhaps sometimes the culture of the place that you're working … I remember my very first placement was at a mental health rehabilitation in-patient unit. Actually, the holistic care there was very much like helping with housing and a lot of social support and helping people, you know, focus on being able to cook themselves a meal as well as very much integral to their health and wellbeing.*” Nursing FG.

*“I think like it depends on the complexity of the condition, because I had a patient at a previous placement with multiple myeloma and there was lots of different MDT teams involved*.” Physiotherapy FG

Nurses perceived that certain work practices such as comprehensive handovers gave an insight into the non-medical aspects such as the patients’ situation and emotion and helped them to do better as patient-centred practitioners.

*“Not all nurses will do this but a lot of nurses will go through all their medical needs, everything, go through their drugs and stuff and then they'll say how they've been in the day. Like, if they've been upset by anything or … It helps me so much because you go with that mindset,”* Nursing FG

d) Students perceived dilemmas due to workplace culture and practices such as patients being treated as cases in learning situations such as ward-rounds. Students, especially Physiotherapists, felt the burden of the workload and found balancing the ideal and realistic commitments challenging. They were constantly prioritising which was frustrating and sometimes disappointing. Nurses stated that they struggled to divide attention between the physical care and spending time with patients.

*“Especially on ward-rounds, when you go around with them they can see all of the patients as more of like a list to get through before a certain amount of time. I suppose, the time pressure and all the - almost they turn it into like tours for all the tests that need to be done for each patient, every single morning. I suppose, it makes it less patient-centred and more like a factory sometimes.*” Medical FG

*“ … like obviously with the limitations of your workload and the number of staff you've got, sometimes you just can't give every patient what is best for them, I think … . You've got to balance what they want but the actual clinical needs as well, and it's trying to find that balance, or as much as possible, within the limitations of the service and everything.”* Physiotherapy FG

*“I think that's my biggest barrier to it is being so concerned with other things that maybe my patient-centred or family-centred care lacks,..”* Nursing FG.

Students concluded that PCC is not always possible in the real world; this sometimes meant establishing a balance between patient’s freewill and their clinical need.

*"I think sometimes it doesn't happen though. I think everyone's purpose here, like all healthcare professionals, is doing what the patient wants to help them get better, but obviously patient-centred practice can't be like the main forefront. The main aim of like people in hospital is to get them out again because of the measures, like costs and the effectiveness, so patient-centred care is going to take a bit of a back burner in certain situations, if that makes sense.*" Physiotherapy FG.

### Integrated findings

#### Discrepancy in patient-centred orientation scores and expressed views

Overall mean scores for all items was 4.0 which is considered clinician-centred orientation rather than patient-centred. This was despite a good theoretical understanding of the concept voiced by all groups of students. Caring scores were the lowest (3.4) and yet students expressed altruistic motives. Aspects of what has been termed as hidden curriculum has been expressed here as factors that affect the development of patient-centred attitudes. The pressures and culture in the NHS have been reflected in this hidden curriculum and the key factor of inadequate mentoring is a good example. Hence it can be interpreted that health professional students have a comprehensive understanding of patient-centred approach and ideal patient-centred motives, but, in the real-world, expression of these motives is restricted.

#### Task-orientation and NHS exposure corrodes patient-centredness

The physiotherapy students’ PPOS scores were significantly lower compared to the medical students in total, caring and sharing components. This was despite having had more placements and having placements in physical rehabilitation which have longer patient contact. Physiotherapy students seemed to be goal-oriented and were in situations where they had to prioritise completion of tasks rather than care for patients (evidenced by Physiotherapy quotes). This was perhaps due to low staffing levels and their responsibility to step up. Their clinician-centred orientations (i.e. low PPOS scores) could be as a result of trying to get through their caseloads. This could be true for the nursing students as well since they too had lower scores despite having had more placements. It can be said that the medical students in their third year were still idealistic due to low exposure to work pressures. This is supported by their views that they can spend more time with patients early in their student years and hence could be less task-oriented.

#### Gender and maturity

SALT students scored higher than physiotherapy and nursing students in all three PPOS scores. Considering this against the background of their gender and maturity, all of them were females (100%) with a higher percentage of mature students (42%). Female gender has previously been shown to be a predictor of patient-centred orientation [36]. However, based on this study, maturity along with its wider experiences and responsibilities could be a key role in influencing patient-centred orientation.

#### Comparison across professions

There were clear similarities within the values of all student groups as highlighted by their altruistic motives. They all reflected a need for caring model of practice. However, differences in their aims of education and learning experiences heavily influenced by the working culture on placements were seen to affect their development as patient-centred practitioners within the four groups. Physiotherapy students were task oriented due to their professional aim of improving patients’ functional independence whilst the medical students focused on developing their biomedical knowledge. Though all groups mentioned lack of role models on placements, nursing students described a dominant clinical supervisory role which impeded their autonomy to carry out patient-centred practice. The extremely busy working environment seemed to have affected the physiotherapy and nursing students’ development as patient-centred practitioners more than the medical and SALT students (who had a smaller number of placements) as reflected by their PPOS scores.

## Discussion

The key findings showed that overall PPOS scores were leaning towards clinician-centred orientation (Table 1). The differences in scores were significant within the four disciplines, especially between the medical and physiotherapy students. Enrollment in medicine and SALT predicted significantly higher total PPOS scores. The focus groups revealed that students understood patient-centredness as holistic care tailored to each person through understanding of individual needs and through building a relationship; their conceptualisation was congruent with literature [28]. Students from all four disciplines identified placement learning as vital and role models from placements as the key factor that could either positively or negatively influence development of patient-centred attributes. They suggested working situations and their status as students caused them dilemmas that shaped their attitudes towards patient-centred care.

To our knowledge, in this era of multidisciplinary working and interprofessional learning, this is the first study that explores and compares patient-centred orientations of students from multiple health professions. Further this study is unique in that we sought to explain the influences for the extent of patient-centred orientation in these students through qualitative data as well as quantitative. There is a preponderance of studies of medical students [37], but, this study brings together a shared understanding of learning to be patient-centred, which is foundational for developing patient-centred education. Further, gaining a shared understanding is vital for interprofessional collaborative activities such as in multidisciplinary meetings to deliver PCC through mechanisms for communication, participation and contribution from different disciplines [38].

More widely, there have been calls to improve patient-centred orientation in the healthcare workforce due to economic and technological developments in Asian countries. However, the PPOS scores of the students in England in this study and elsewhere is higher than Asian medical students (3.90) [37] (3.40) [39], but lower than the medical students training in America (4.76–4.84) [40]. This has been attributed to the doctor-centred approach to care in Asian countries [39, 41, 42]. Research suggests that patients lean towards family centred care in these countries [43]. Hence, while designing patient-centred education in different countries, cultural influences should be considered.

In our study medical students scored better compared to nursing and physiotherapy students despite the fact they were still halfway through their education and had fewer placement experiences. It is known that professionals are more patient-centred early in their career compared to in their later years [8, 24]. The third year of education has been revealed as the turning point when medical students tend to step away from patient-centred attitudes [44]. Perhaps, medical students in this study showed better patient-centred orientation compared to other groups due to lesser influence of the hidden curriculum since they had less exposure to the NHS working practices and culture. Meanwhile the nursing and physiotherapy students in this study had faced high workload and demanding responsibilities and had moved away from patient-centred orientation. Elsewhere, nursing students have been shown to score higher in caring than sharing [24]. In physiotherapy, students’ understanding of the patient-centred approach involved being task oriented and getting patients to be compliant to achieve goals. Their approach to care was pragmatic and suggested PCC might not always be possible. This suggests that the educational approach to be patient-centred cannot be a one size fits all approach for different caring and therapy professions. Just as patient-centred care is individualistic to each person, ways of adopting principles should be differently designed for professions that deliver physical care and different for other therapy professions that devolve ownership of health to patients.

Females have previously been shown to be more patient-centred and scored higher in caring and total scores compared to male clinicians [45]. Perhaps the higher scores of the SALT group in this study reflects the gender composition of this group with 100% of them being females. Moreover, there was a higher percentage of mature students within the SALT group who were identified to be more patient-centred in other studies [36, 45]. It is possible that mature students choose to do health professional courses due to intrinsic motivation gained from life experiences. Mature students have also been shown to be more empathetic [46]. Hence their patient-centred attitudes are embedded in their personality rather than balancing the development of these attitudes along with professional and medical skills.

Previous studies showed that those students with wider work experience showed better patient-centred orientation [39, 45] contrary to the findings of this study. The physiotherapy and nursing students who had a greater number of placements by the end of third year did not show high patient-centred orientation compared to medical and SALT students who had fewer placement experiences by this point. Perhaps not having positive role models on placements, inadequate support from mentors, influence of ‘hidden curriculum’ might account for work experience not enabling their patient-centred attitudes [26]. This is supported by data from the current study’s focus groups which highlight the negative influence of mentoring in all four groups resulting in students being unable to resist cultural influences and instead followed prevalent practices. Hence it is important to turn the negative influence of the socialisation process [47] around by creating awareness and training mentors in this aspect [27]. It is like minded patient-centred clinical educators who can reduce the current gap in conflicts in learning about PCC at University and the actual practice environment [27].

Practice placement type did not predict their patient-centred orientation in this study. This is interesting as Kitson et al’s (2013) review has identified that care context maybe a key factor in the level of PCC in practice due to barriers such as time and resources and philosophical differences in approach to care in different settings [28]. In Nigeria psychiatry doctors were found to be most patient centred compared to those working in internal and family medicine [48]. The authors had suggested that this could be due to low mortality rates in these patient groups, therefore patients were in the care system for long periods. Moreover, consultation times were longer, 45 mins for each psychiatry patient. Even though this could hold true for UK practice, medical students seem to have observed differently. In the FG they reported that mental health patients were not given adequate information due to assumption of reduced mental capacity. Whether this observed learning influences their future practice in mental health or other areas with reduced mental capacity needs to be explored.

### Limitations

Conducting the survey close to the end of the year and close to exam period might have affected the response rate for medical and nursing students. This and the study taking place in two Universities in the West Midlands might reduce the generalisability of findings to wider Universities. A sample size was not estimated for the survey due the exploratory nature of the study. Other factors such as spirituality [26], cultural background, specific training elements could have contributed to the patient-centred orientation but were not studied in this research. Regarding the FG methodology, no repeat groups were arranged to seek data saturation as the ongoing analysis showed repetition and overlap within data from different groups. Member checking was not possible as most of the students left University following completion of their studies.

Future research should investigate other variables that influence patient-centred orientation in students in a bigger sample size across a wider geographical area. It is also important to explore aspects within the curriculum that influence the development of patient-centred attributes. This will help build curriculum that is based on students’ values and needs. Since the role of mentoring in developing patient-centred attributes is heavily highlighted from this study, future research should focus on how educators and clinicians perceive their role and contribution in this aspect. Perhaps placement education systems should provide patient-centredness training to educators based on the study’s findings to enhance educators’ contribution towards developing patient-centred healthcare workforce.

## Conclusion

Despite increasing focus on developing patient-centredness in health care practitioners in the past few decades, there is limited research on how students from different professions working together in a team compare to each other in patient-centred orientations and what their shared understanding of this concept is. This mixed methods study explored student perceptions from different disciplines. In this geographical area, medical students and SALT students seemed to score higher in their levels of patient-centred orientations compared to physiotherapy and nursing students. A key challenge identified by all the four disciplines for their development as patient-centred professionals was the lack of role models which necessitates further research and patient-centred training of clinical educators. The hidden curriculum due to the pressures of the NHS has been suggested to negatively influence certain professional disciplines whereas the professional disciplines with less exposure to the NHS pressures had better patient-centred orientations. This raises questions about our clinical training models. Future research should look at how the influencing factors can be modified and build better ways of training and assessment especially in the clinical placements as highlighted by this study.

## Additional files

Additional file 1: Clinical placements categorised under specialisms (DOCX 13 kb)

Additional file 2: Patient- Centred Care- Question guide for Focus Group –Version 1-dated 20.03.17 (DOCX 13 kb)

Additional file 3: Multiple Regression Analysis (DOCX 17 kb)

## Data Availability

The datasets used and/or analysed during the current study are available from the corresponding author on reasonable request.

## Abbreviations

ANOVA: Analysis of Variance
CI: Confidence Interval
FG: Focus group
MBChB: Bachelor of Medicine and Bachelor of Surgery
NHS: National Health Service
PCC: Patient-centred care
PPOS: Patient-Practitioner Orientation Scale
SALT: Speech and Language Therapy
SD: Standard Deviation
SPSS: Statistical Package for the Social Sciences

## References

[1] Francis R. Report of the mid Staffordshire NHS Foundation trust public inquiry. London; 2013.

[2] Keogh B. Review into the quality of care and treatment provided by 14 hospital trusts in England: overview report. London; 2013.

[3] Kennedy I. Bristol Royal Infirmary Inquiry. Learning from Bristol:the report of the public inquiry into children’s heart surgery at the Bristol Royal Infirmary 1984–1995. London; 2001.

[4] Department of Health (DoH) (2010). Equity and excellence: liberating the NHS.

[5] Department of Health (DoH) (2013). Patients first and foremost-the initial government response to the report of the mid Staffordshire NHS Foundation trust public inquiry.

[6] Department of Health (2013). The NHS constitution.

[7] Mead M, Bower P (2000). Patient-centredness: a conceptual framework and review of the empirical literature. Soc Sci Med.

[8] Krupat E, Rosenkranz SL, Yeager CM, Barnard K, Putnam S, Inui TS (2000). The practice orientations of physicians and patients: the effect of doctor–patient congruence on satisfaction. Patient Education & Counseling.

[9] Rathert C, Wyrwich MD, Boren SA (2012). Patient-centered care and outcomes: a systematic review of the literature. Med Care Res Rev.

[10] Stewart M, Brown JB, Donner A, McWhinney IR, Oates J, Weston WW (2000). The impact of patient-centered care on outcomes. J Fam Pract.

[11] Office of Patient Centered Care and Cultural Transformation. Whole health – It’s all about you; a report by the VHA Office of Patient Centered Care and Cultural Transformation. Washington: Department of Veterans Affairs; 2017.

[12] Dixon-Woods Mary, Baker Richard, Charles Kathryn, Dawson Jeremy, Jerzembek Gabi, Martin Graham, McCarthy Imelda, McKee Lorna, Minion Joel, Ozieranski Piotr, Willars Janet, Wilkie Patricia, West Michael (2013). Culture and behaviour in the English National Health Service: overview of lessons from a large multimethod study. BMJ Quality & Safety.

[13] Arenson C, Umland E, Collins L, Kern SB, Hewston LA, Jerpbak C (2015). The health mentors program: three years experience with longitudinal, patient-centered interprofessional education. Journal of Interprofessional Care.

[14] Yasui H, Abe K, Amioka K, Ishigro S, Norose T, Sakurai S, et al. Does 'Interprofessional Education' in the Uni-professional setting improve students understanding of patient-Centred care? Med Educ 2014;48:12- 1p.

[15] Klocko DJ, Krumwiede KH, Olivares-Urueta M, Williamson JW (2012). Development, implementation, and short-term effectiveness of an Interprofessional education course in a School of Health Professions. J Allied Health.

[16] Boudreau JD, Cassell EJ, Fuks A (2007). A healing curriculum. Med Educ.

[17] Roskell C, White D, Bonner C (2012). Developing patient-centred care in health professionals: reflections on introducing service-learning into the curriculum. Int J Ther Rehabil.

[18] Rapport MJ, Rodriguez J, Bade M (2010). Use of a community volunteer program to develop value for patient-centered care in physical therapist professional education. Journal of Physical Therapy Education.

[19] Smith Stephen R., Cookson John, Mckendree Jean, Harden Ronald M. (2007). Patient-centred learning—back to the future. Medical Teacher.

[20] Archer E, Bezuidenhout J, Kidd M, Van Heerden BB (2014). Making use of an existing questionnaire to measure patient-centred attitudes in undergraduate medical students: a case study. Afr J Health Prof Educ.

[21] Bosma Hans, Diederiks Jos, Scherpbier Albert, Van Eijk Jacques (2010). A gender-specific evaluation of a care-oriented curricular change in a Dutch medical school. Medical Teacher.

[22] Haidet P, Dains JE, Paterniti DA, Hechtel L, Chang T, Tseng E (2002). Medical student attitudes toward the doctor–patient relationship. Med Educ.

[23] Howe A (2001). Patient-centred medicine through student-centred teaching: a student perspective on the key aspects of community-based learning in undergraduate medical education. Med Educ.

[24] Grilo AM, Santos MC, Rita JS, Gomes AI (2014). Assessment of nursing students and nurses' orientation towards patient-centeredness. Nurse Educ Today.

[25] Haidet P, Dains JE, Paterniti DA, Hechtel L, Chang T, Tseng E (2002). Medical student attitudes toward the doctor-patient relationship. Med Educ.

[26] Tsimtsiou Z, Kerasidou O, Efstathiou N, Papaharitou S, Hatzimouratidis K, Hatzichristou D (2007). Medical students' attitudes toward patient-centred care: a longitudinal survey. Med Educ.

[27] Hafferty FW, Gaufberg EH, O'Donnell JF (2015). The role of the hidden curriculum in "on doctoring" courses. AMA J Ethics.

[28] Kitson A, Marshall A, Bassett K, Zeitz K (2013). What are the core elements of patient-centred care? A narrative review and synthesis of the literature from health policy, medicine and nursing. J Adv Nurs.

[29] Teddlie C, Tashakkori A (2009). Foundations of mixed methods research: integrating quantitative and qualitative approaches in the social and behavioral sciences.

[30] Kidd PS, Parshall MB (2000). Getting the focus and the group: enhancing analytical rigor in focus group research. Qual Health Res.

[31] Bengtsson M (2016). How to plan and perform a qualitative study using content analysis. NursingPlus Open.

[32] Ross EF, Haidet P (2011). Attitudes of physical therapy students toward patient-centered care, before and after a course in psychosocial aspects of care. Patient Educ Couns.

[33] Dockens AL, Bellon-Harn ML, Manchaiah V (2016). Preferences to patient-centeredness in pre-service speech and hearing sciences students: a cross-sectional study. J Audiol Otol.

[34] Field A (2013). Discovering statistics using IBM SPSS statistics.

[35] Ivankova N, Creswell JW, Stick LS (2006). Using mixed-methods sequential explanatory design: from theory to practice. Field Methods.

[36] Wahlqvist M, Gunnarsson RK, Dahlgren G, Nordgren S (2010). Patient-centred attitudes among medical students: gender and work experience in health care make a difference. Med Teach.

[37] Hur Y, Cho AR, Choi CJ (2017). Medical students' and patients' perceptions of patient-centred attitude. Korean J Med Educ.

[38] Fox A, Reeves S (2015). Interprofessional collaborative patient-centred care: a critical exploration of two related discourses. Journal of Interprofessional Care..

[39] Ahmad W, Krupat E, Asma Y, Fatima NE, Attique R, Mahmood U (2015). Attitudes of medical students in Lahore, Pakistan towards the doctor-patient relationship. PeerJ..

[40] Al-Bawardy R, Blatt B, Al-Shohaib S, Simmens SJ (2009). Cross-cultural comparison of the patient-centeredness of the hidden curriculum between a Saudi Arabian and 9 US medical schools. Medical education online.

[41] Tor PC (2001). New challenges facing the doctor–patient relationship in the next millennium. Singap Med J.

[42] Shankar RP, Piryani RM (2009). Medical education and medical educators in South Asia –a set of challenges. Journal of the College of Physicians and Surgeons Pakistan.

[43] Slingsby BT (2005). Professional approaches to stroke treatment in Japan: a relationship-centred model. J Eval Clin Pract.

[44] Hojat M, Vergare MJ, Maxwell K, Brainard G, Herrine SK, Isenberg GA (2009). The devil is in the third year: a longitudinal study of erosion of empathy in medical school. Academic medicine : journal of the Association of American Medical Colleges.

[45] Lumma-Sellenthin A (2012). Students’ attitudes towards learning communication skills: correlating attitudes,demographic and metacognitive variables. Int J Med Educ.

[46] Nunes P, Williams S, Sa B, Stevenson K (2011). A study of empathy decline in students from five health disciplines during their first year of training. Int J Med Educ.

[47] Hafferty FW (1998). Beyond curriculum reform: confronting medicine's hidden curriculum. Academic medicine : journal of the Association of American Medical Colleges..

[48] Tajudeen Abiola ATA, Udofia O, Baguda A, Habib ZG (2016). Patient-centeredness: a comparison of doctors’ orientation by specialty in a teaching Hospital in Nigeria. Journal of Earth, Environment and Health Sciences.

