# Supplementary material for: Patient-centred orientation of students from different healthcare disciplines, their understanding of the concept and factors influencing their development as patient-centred professionals: a mixed methods study
Source: BMC Med Educ. 2019 Sep 11;19:347. doi: 10.1186/s12909-019-1787-4 (PMC6737623; doi:10.1186/s12909-019-1787-4)
Supplement: Supplementary file 1 — Clinical placements categorised under specialisms (DOCX 13 kb) [file 12909_2019_1787_MOESM1_ESM.docx]

**Additional file 1: Clinical placements categorised under specialisms**

| Acute and Specialisms | Community and Hospices | Paediatric | Mental Health | Physical rehabilitation |
| --- | --- | --- | --- | --- |
| Burns  Medicine  Critical-care  stroke  MSK  Respiratory  Surgery  OPD  Acute Hospital -Adults  Womens-health  Oncology  Trauma  Orthopaedic  Neuro  Elderly care | Community  Community Nursing  Palliative  Hospice Adults Child | Paediatrics  Acute hospital for  Children  Nursery  School | Mental health  Inpatient Mental health  Community Mental health | Rehabilitation  Hydrotherapy  Intermediate-care  Regional Posture and Mobility clinic  FES |
