# Supplementary material for: Patient-centred orientation of students from different healthcare disciplines, their understanding of the concept and factors influencing their development as patient-centred professionals: a mixed methods study
Source: BMC Med Educ. 2019 Sep 11;19:347. doi: 10.1186/s12909-019-1787-4 (PMC6737623; doi:10.1186/s12909-019-1787-4)
Supplement: Supplementary file 2 — Patient- Centred Care- Question guide for Focus Group –Version 1-dated 20.03.17 (DOCX 13 kb) [file 12909_2019_1787_MOESM2_ESM.docx]

**Additional file 2:**

**Patient- Centred Care- Question guide for Focus Group –Version 1-dated 20.03.17**

1. What do you understand by the term patient-centred care?
2. Is this an important aspect of healthcare? If so, why? Or why not?
3. As a student what challenges do you face in delivering patient-centred care?
4. As a student what enables you to deliver patient-centred care?
5. Where did you learn about aspects of patient-centred care and how to deliver it?
6. Can you tell us how you are taught within your course about what is patient-centred care and how it can be applied in practice?
7. Can you tell us how you are taught on your clinical placements about what is patient-centred care and how it can be applied in practice?
8. What do you think is the best way to teach principles and delivery of patient-centred care?
