# Supplementary material for: Patient-centred orientation of students from different healthcare disciplines, their understanding of the concept and factors influencing their development as patient-centred professionals: a mixed methods study
Source: BMC Med Educ. 2019 Sep 11;19:347. doi: 10.1186/s12909-019-1787-4 (PMC6737623; doi:10.1186/s12909-019-1787-4)
Supplement: Supplementary file 3 — Multiple Regression Analysis (DOCX 17 kb) [file 12909_2019_1787_MOESM3_ESM.docx]

**Additional file 3:**

**Multiple Regression Analysis**

|  | **CARING** | | | | **SHARING** | | | | | **TOTAL SCORES** | | | | |
| --- | --- | --- | --- | --- | --- | --- | --- | --- | --- | --- | --- | --- | --- | --- |
|  | **Β** | **SE** | **T value** | **P value** | **β** | **SE** | **T value** | **P value** | **β** | | **SE** | **T value** | **P value** |  |
| **(Intercept)** | 32.03 | 2.053 | 15.604 | 0 | 35.02 | 2.282 | 15.345 | 0 | 32.03 | | 2.053 | 15.604 | 0 |  |
| **ProgPhysiotherapy** | -1.675 | 1.375 | -1.218 | 0.225 | 0.106 | 1.529 | 0.069 | 0.945 | -1.675 | | 1.375 | -1.218 | 0.225 |  |
| **ProgMedicine** | 3.462 | 1.578 | 2.193 | 0.029 | 3.012 | 1.755 | 1.716 | 0.088 | 3.462 | | 1.578 | 2.193 | **0.029** |  |
| **ProgSALT** | 4.206 | 1.493 | 2.817 | **0.005** | 2.743 | 1.66 | 1.653 | 0.1 | 4.206 | | 1.493 | 2.817 | **0.005** |  |
| **Sex M** | -1.627 | 1.017 | -1.6 | 0.111 | -1.74 | 1.13 | -1.539 | 0.125 | -1.627 | | 1.017 | -1.6 | 0.111 |  |
| **Mature student status** | -1.259 | 0.88 | -1.432 | 0.154 | 0.236 | 0.978 | 0.242 | 0.809 | -1.259 | | 0.88 | -1.432 | 0.154 |  |
| **Paediatric** | -0.776 | 1.039 | -0.746 | 0.456 | -0.278 | 1.156 | -0.241 | 0.81 | -0.776 | | 1.039 | -0.746 | 0.456 |  |
| **Mental health** | 0.119 | 1.102 | 0.108 | 0.914 | 0.402 | 1.225 | 0.328 | 0.743 | 0.119 | | 1.102 | 0.108 | 0.914 |  |
| **Physical rehabilitation** | 1.725 | 1.404 | 1.229 | 0.221 | -1.817 | 1.561 | -1.164 | 0.246 | 1.725 | | 1.404 | 1.229 | 0.221 |  |
| **Acute hospitals and specialisms** | 1.421 | 1.323 | 1.074 | 0.284 | 2.2 | 1.471 | 1.495 | 0.137 | 1.421 | | 1.323 | 1.074 | 0.284 |  |
| **Community and Hospices** | 0.641 | 0.722 | 0.888 | 0.376 | 0.826 | 0.803 | 1.028 | 0.305 | 0.641 | | 0.722 | 0.888 | 0.376 |  |
|  | Adj $R^{2}$ = 16.3 % | | | | Adj $R^{2}$ = 5.5 % | | | | Adj $R^{2}$ = 15.3 % | | | | |  |
